# TACE Empowers Immune Checkpoint Inhibitors and Tyrosine Kinase Inhibitors in Unresectable HCC: A Multicenter Retrospective Study

**DOI:** 10.7150/jca.112706

**Published:** 2025-06-12

**Authors:** Yu Lei, Yaowei Bai, Xiatong Bai, Jiacheng Liu, Bo Sun, Wenlong Wu, Xiaoling Zhi, Yang Su, Hongsen Zhang, Chuansheng Zheng

**Affiliations:** 1 Department of Radiology, Union Hospital, Tongji Medical College, Huazhong University of Science and Technology, Wuhan 430022, China.; 2 Hubei Provincial Clinical Research Center for Precision Radiology & Interventional Medicine, Wuhan 430022, China.; 3 Hubei Province Key Laboratory of Molecular Imaging, Wuhan 430022, China.

**Keywords:** transarterial chemoembolization, TKI, ICI, combination therapy, hepatocellular carcinoma

## Abstract

**Purpose**: The aim of this multicenter retrospective study was to evaluate the efficacy and safety of transarterial chemoembolization (TACE) combined with tyrosine kinase inhibitors (TKI) and immune checkpoint inhibitors (ICI) in treating advanced hepatocellular carcinoma (HCC) compared to treatment with TKI and ICI alone.

**Methods**: The study included 286 patients with advanced HCC, of which 210 were treated with TACE, TKI, and ICI (TACE+T+I group) and 76 with TKI and ICI alone (T+I group). Progression-free survival (PFS), overall survival (OS), overall response rate (ORR), and disease control rate (DCR) were assessed. A nomogram was developed to stratify patients into high-risk and low-risk groups based on their one-year and two-year survival probabilities.

**Results**: Patients in the TACE+T+I group demonstrated significantly longer PFS (8.4 months vs. 4.0 months, Log-rank *P* = 0.0016) and median OS (14.5 months vs. 10.0 months, Log-rank *P* < 0.0001) compared to the T+I group. Additionally, the TACE+T+I group had a higher ORR (56.7% vs. 21.1%, *P* = 0.002) and DCR (84.3% vs. 72.4%, *P* = 0.023). Both groups exhibited good tolerance to adverse events. A nomogram incorporating factors such as therapeutic strategy, prothrombin time (PT), age, and tumor size effectively categorized patients into low- and high-risk groups with notably different survival outcomes.

**Conclusion**: These findings suggest that TACE combined with TKI and ICI significantly improved survival outcomes and showed good safety compared to TKI and ICI alone in the treatment of advanced HCC.

## Introduction

Liver cancer is the sixth most prevalent form of cancer globally and is the third leading cause of cancer-related fatalities 1, 2. Typically, early-stage liver cancer presents with subtle symptoms, leading to a majority of diagnoses at advanced stages, where surgical intervention may no longer be an option. As the most common type of liver cancer, hepatocellular carcinoma (HCC) patients face poor prognosis due to rapid tumor growth, intrahepatic spread, and distant metastasis. According to the current Barcelona Clinic Liver Cancer (BCLC) staging system, systemic therapies such as tyrosine kinase inhibitors (TKI) and immune checkpoint inhibitors (ICI) are the recommended treatments for these patients in the middle to advanced stages 3, 4.

TKI drugs, which include sorafenib 5 and Lenvatinib 6, are designed to target and inhibit specific tyrosine kinase receptors on cancer cells, thus impeding the formation of new blood vessels and tumor growth. ICI are a type of drugs that activate the patient's immune system to recognize and attack tumor cells. They target the PD-1/PD-L1 and CTLA-4 immune checkpoints, enhancing T-cell activation and proliferation, and restoring T-cell anti-tumor activity 7-10. These drugs offer more treatment options for patients with advanced HCC, potentially extending their survival and enhancing their quality of life 11,12. Nonetheless, certain research indicates that the overall survival (OS) with TKI monotherapy is only 6.5-8.1 months 13,14. In the case of ICI administered alone, such as pembrolizumab and nivolumab, the therapeutic impact is modest, with an objective response rate (ORR) of only 14.7-20% 15,16.

Transarterial chemoembolization (TACE) is also a recommended and commonly used treatment for advanced HCC 3,17-19. TACE induces tumor cell death, releasing tumor antigens and promoting immune cell infiltration, which synergizes with ICI 20,21. Additionally, TKI can inhibit the elevated VEGF expression after TACE 22, providing theoretical support for the combination of TACE and TKI. A recent meta-analysis pointed out that the triple therapy of TACE, TKI, and ICI offers enhanced tumor response and improved survival outcomes for patients with advanced HCC, and has the highest disease control rate (DCR) compared to other dual therapies 23. Similarly, the phase III LEAP-012 trial indicated that the combination of pembrolizumab and lenvatinib with TACE can reduce the risk of tumor progression and mortality by 34% 24. However, debate persists regarding whether the combined therapy truly offers superior tumor treatment outcomes and translates into tangible survival advantages for patients.

In light of the above background, this study aimed to analyze extensive multicenter data to evaluate the effectiveness and safety of a combined therapy involving TACE, TKI, and ICI, compared to standalone TKI and ICI treatments for patients with advanced HCC.

## Materials and Methods

### Patient selection

A retrospective study was conducted on 286 patients with advanced HCC who received treatment with TKI and ICI, either alone or in combination with TACE, at three hospitals: Wuhan Union Hospital, the First Affiliated Hospital of Henan University of Science and Technology, and the First Affiliated Hospital of Zhengzhou University, from January 2021 to October 2024. Among them, Wuhan Union Hospital enrolled 216 patients, the First Affiliated Hospital of Henan University of Science and Technology enrolled 15 patients, and the First Affiliated Hospital of Zhengzhou University enrolled 55 patients.

The inclusion criteria for patients included: (1) age over 18 years old; (2) diagnosis of advanced HCC by medical imaging techniques or biopsy according to the guidelines of the American Association for the Study of Liver Diseases (AASLD); (3) tumor localized to a limited area without evidence of extrahepatic metastasis; (4) Child-Pugh A or B class; (5) ECOG performance status of 0-2; (6) expected survival time of more than 3 months. Key exclusion criteria were as follows: (1) diffuse HCC or tumor burden exceeding 70% of the entire liver; (2) previous treatments such as surgery or liver transplantation; (3) other serious malignant diseases; (4) uncontrolled ascites; (5) severe coagulation dysfunction, renal insufficiency, and uncorrectable cardiopulmonary dysfunction; (6) incomplete medical information or loss of follow-up.

**Figure 1:**
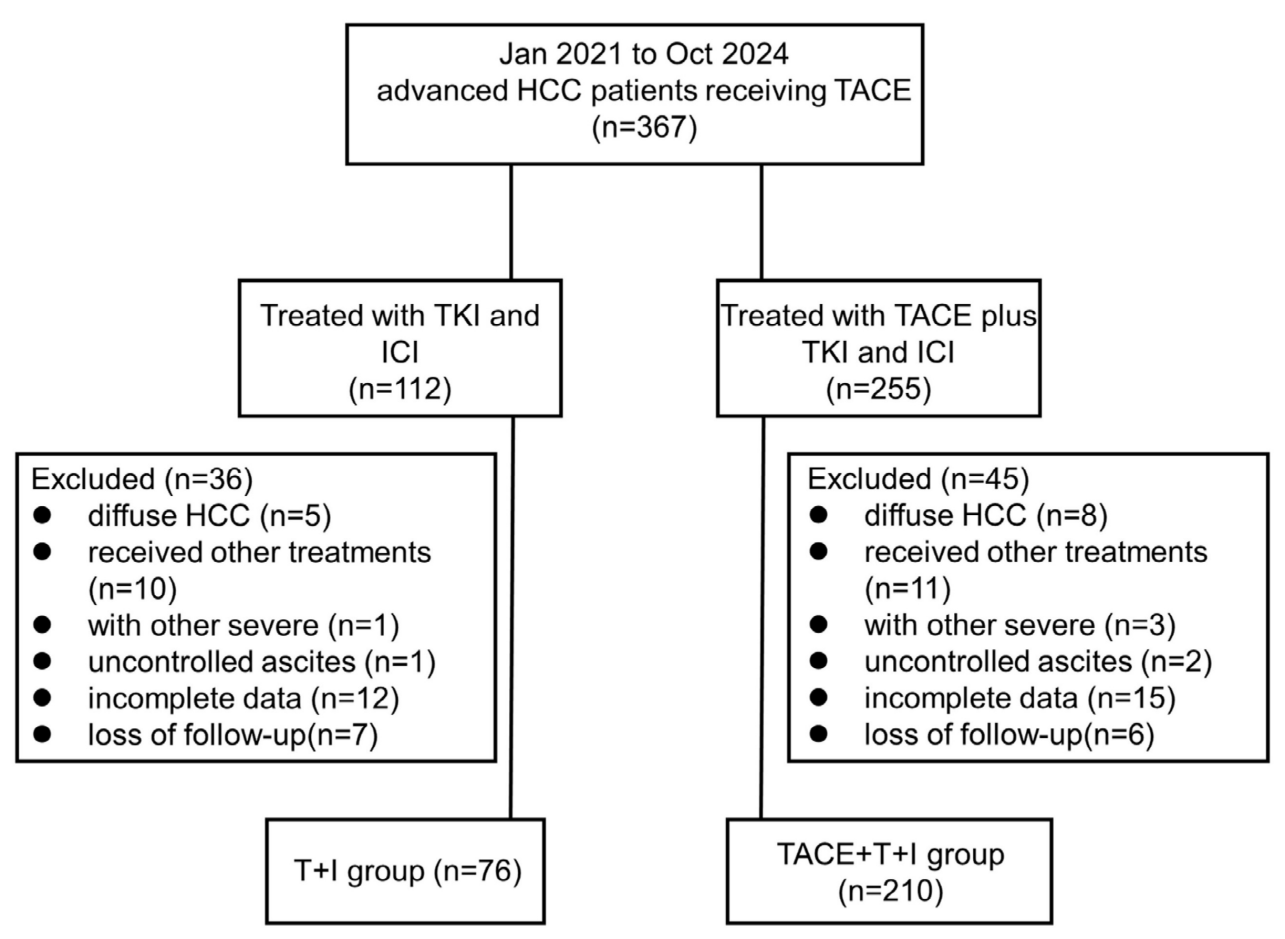
Patient flow chart. HCC, hepatocellular carcinoma; TACE+T+I, transarterial chemoembolization combined with tyrosine kinase inhibitor and immune checkpoint inhibitor group; T+I, tyrosine kinase inhibitor combined with immune checkpoint inhibitor group; TACE, transarterial chemoembolization; ICI, immune checkpoint inhibitor; TKI, tyrosine kinase inhibitor.

Ultimately, 210 patients received TACE combined with TKI and ICI treatment were included in the combined treatment group (TACE+T+I group), and 76 patients received only TKI and ICI treatment were included in the T+I group. The inclusion and exclusion process of this study is described in Fig. 1.

This study was approved by the Institutional Review Board of Wuhan Union Hospital, Tongji Medical College, Huazhong University of Science and Technology and conducted in accordance with the ethical principles of the World Medical Association's Declaration of Helsinki. Due to the retrospective nature of the study and the use of anonymized clinical data, written informed consent was revoked by IRB of Wuhan Union Hospital, Tongji Medical College, Huazhong University of Science and Technology.

### TACE procedure

The TACE procedure was performed as described by Wang et al. 25, conducted by interventional radiologists with more than 10 years of clinical experience under local anesthesia. A 5-French catheter (COOK) was inserted through the femoral artery and advanced to the celiac and superior mesenteric arteries for angiography to determine the number, size, location, and feeding arteries of the tumor. Then, a 2.7-French microcatheter (Terumo, Tokyo, Japan) was superselectively inserted into the tumor feeding artery to perform embolization treatment. Ten milligrams of epirubicin were dissolved in 2 ml of contrast medium, and a mixture of lipiodol (10 ml) and epirubicin solution was prepared using a three-way stopcock. In cases of HCC with low-flow shunts where iodized oil did not reach non-target regions, conventional TACE was conducted under fluoroscopic guidance. Conversely, when iodized oil did spread to non-target areas, embolization was performed using particles (Embosphere microspheres, sized 300-500 μm or 500-700 μm) to obstruct the shunts. Typically, the endpoint of TACE treatment was to achieve complete occlusion of the portal vein and tumor feeding branches. Following TACE, follow-up enhanced CT scans or MRIs were conducted every three weeks. TACE was repeated dependent on the tumor's status (recurrence or residual presence) and the patient's general condition.

### Systemic therapy

ICI was administered intravenously the day after TACE, with a treatment cycle of 3 weeks. The fixed doses for camrelizumab, tislelizumab, and sintilimab were 200mg; the dose for atezolizumab was 1200mg; and the dose for bevacizumab was 15mg/kg of body weight. TKI was taken orally intermittently (250 mg for apatinib; 200 mg for donafenib; 8 mg for lenvatinib). TACE was temporarily stopped in the following situations: disease progression, intolerable adverse events, patients meeting the conditions for another treatment option (such as surgical resection), or patients withdrawing consent. Furthermore, should technical challenges arise during repeating TACE procedures, such as stenosis or occlusion of the tumor-feeding arteries, or if the patient's clinical profile contraindicates further TACE, the study treatment would be suspended. The cessation of treatment depended on multiple factors, including disease progression, death, intolerable adverse events, patient withdrawal of consent, or changes in treatment plans.

### Data collection

All clinical and laboratory data before the first TACE treatment for all patients were collected. Clinical information included age, gender, Eastern Cooperative Oncology Group (ECOG) score, Child-Pugh score, BCLC classification, cirrhosis, hepatitis B virus (HBV) infection, tumor biomarkers, tumor size, and tumor number. Laboratory indicators included red blood cell (RBC) count, white blood cell (WBC) count, hemoglobin (Hb), platelet count (PLT), neutrophil percentage, lymphocyte percentage, total bilirubin (TBIL), albumin (ALB), alanine aminotransferase (ALT), aspartate aminotransferase (AST), alkaline phosphatase (ALP), total protein (ALB), creatinine (Cr), uric acid (UA), total bile acids (TBA), prothrombin time (PT), and international normalized ratio (INR).

### Assessment

Throughout the study, blood routine and safety evaluations were performed following each TACE procedure. To assess tumor response, contrast-enhanced CT scans were performed consecutively. Tumor response was classified according to the modified Response Evaluation Criteria in Solid Tumors (mRECIST) 26, including complete response (CR), partial response (PR), stable disease (SD), and disease progression (PD).

The primary endpoints of the study were OS and progression-free survival (PFS). OS was defined as the time interval from the first treatment to the date of death or the last follow-up (if the patient's death was not recorded). PFS was defined as the time interval from the first treatment to the date of tumor progression based on mRECIST, death from any cause, or the last follow-up (if progression or death was not recorded). The secondary endpoints of the study were ORR, DCR, and adverse events (AEs). ORR was defined as CR+PR and DCR was defined as CR+PR+SD. Adverse events were recorded according to the National Cancer Institute Common Terminology Criteria for Adverse Events (NCI CTCAE; version 5.0).

### Statistical analysis

Descriptive statistics were used to summarize patient characteristics and their laboratory values, presented as numbers (percentages) or mean ± standard deviation. The Student's t-test was used for continuous variables, the Wilcoxon rank-sum test for variables that did not meet the criteria for normal distribution or for ordered variables, and the chi-square test or Fisher's exact test were applied to compare group differences.

Kaplan-Meier analysis was applied to estimate survival curves, with log-rank tests employed for comparing survival outcomes between groups. To determine factors that impact patient outcomes, both univariate and multivariate analyses with the Cox proportional hazards model were conducted to evaluate predictors of PFS and OS. Variables with a P value < 0.1 in univariate analysis were considered potential predictors and were included in the multivariate analysis for further analysis. The R software's rms package was used to construct a nomogram.

Statistical significance was determined by a P value <0.05. The R software (version 4.3.0) and SPSS (version 24.0) were used for statistical analysis.

## Results

### Patient characteristics

From January 2021 to October 2024, a total of 286 patients were enrolled in this study and were divided into the TACE+T+I group (n = 210) and the T+I group (n = 76). The TKIs used by all patients included apatinib, donafenib, and lenvatinib, while the ICIs included camrelizumab, tislelizumab, sintilimab, atezolizumab, and bevacizumab.

The baseline characteristics of all patients are shown in Table 1. In these patients, the majority were male (TACE+T+I group: 86.7%; T+I group: 82.9%; *P* = 0.540), mostly had HBV infection (TACE+T+I group: 81.4%; T+I group: 77.6%; *P* = 0.763), mostly suffered from cirrhosis (TACE+T+I group: 88.6%; T+I group: 86.8%; *P* = 0.847), and most were at the BCLC stage C (TACE+T+I group: 84.3%; T+I group: 76.3%; *P* = 0.176). Overall, patients were well balanced across all variables. The follow-up termination time was October 2024.

### Comparison of PFS between groups

**Figure 2:**
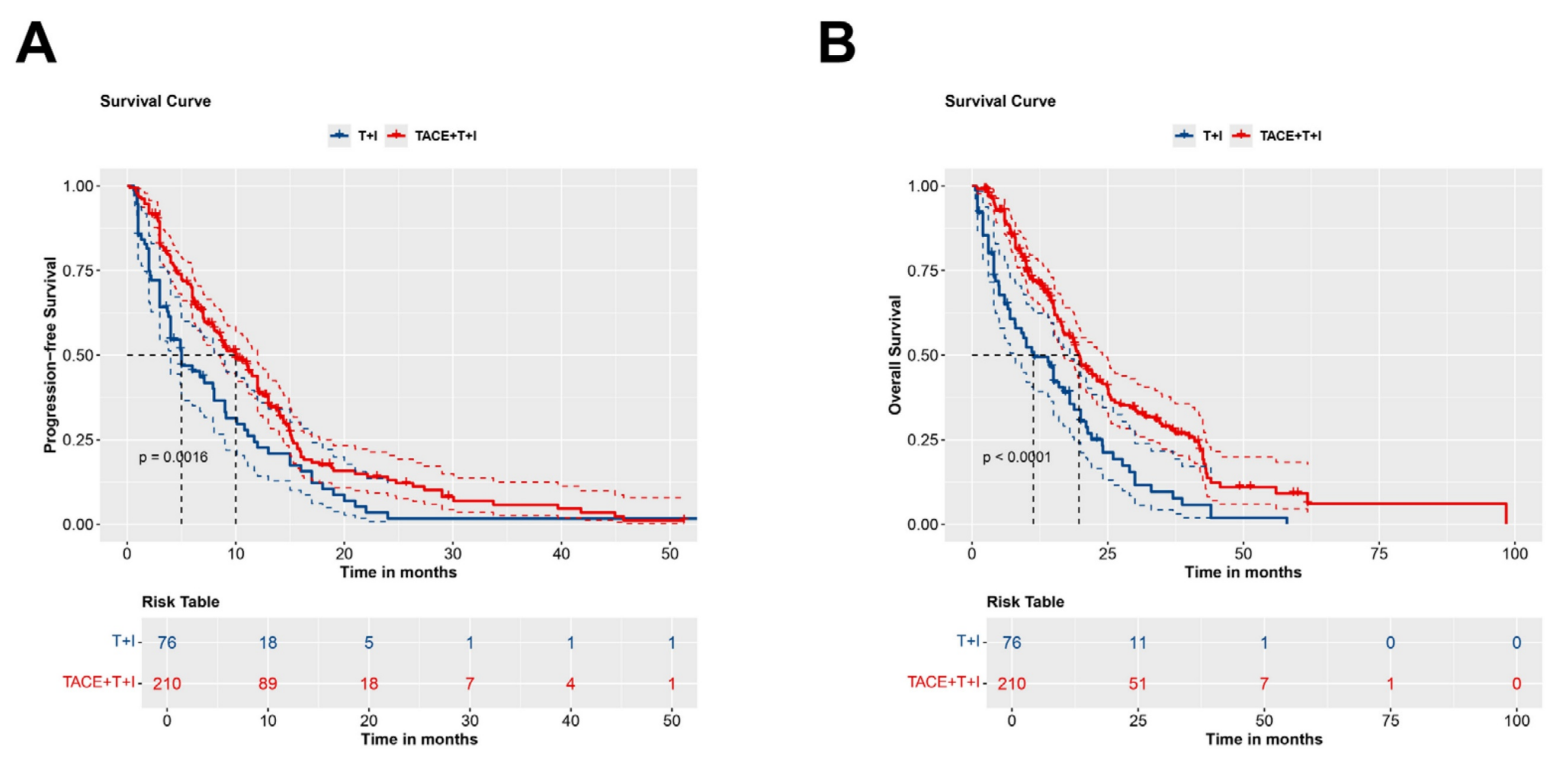
Kaplan-Meier curves for PFS (A) and OS (B) in patients receiving ICI and TKI treatment with/without TACE. TACE+T+I, transarterial chemoembolization combined with tyrosine kinase inhibitor and immune checkpoint inhibitor group; T+I, tyrosine kinase inhibitor combined with immune checkpoint inhibitor group; TACE, transarterial chemoembolization; ICI, immune checkpoint inhibitor; TKI, tyrosine kinase inhibitor.

The median PFS was 8.4 months (Interquartile Range (IQR): 4.2-13.0 months) in the TACE+T+I group and 4.0 months (IQR: 2.0-9.0 months) in the T+I group. Fig. 2A presents the Kaplan-Meier survival curves for PFS in the two groups. The curves demonstrated a significant difference between the groups (Log-rank *P* = 0.0016), indicating that patients in the TACE+T+I group achieved significantly longer PFS compared to those in the T+I group.

### Comparison of OS between groups

The median OS was 14.5 months (IQR: 8.6-24.6 months) in the TACE+T+I group and 10.0 months (IQR: 4.0-20.0 months) in the T+I group. Fig. 2B illustrates the Kaplan-Meier curves for OS in the two groups. A significant difference is observed between two groups, highlighting that patients receiving TACE+T+I therapy exhibited substantially longer OS than those treated with T+I alone (Log-rank *P* < 0.0001).

### Tumor response

**Figure 3:**
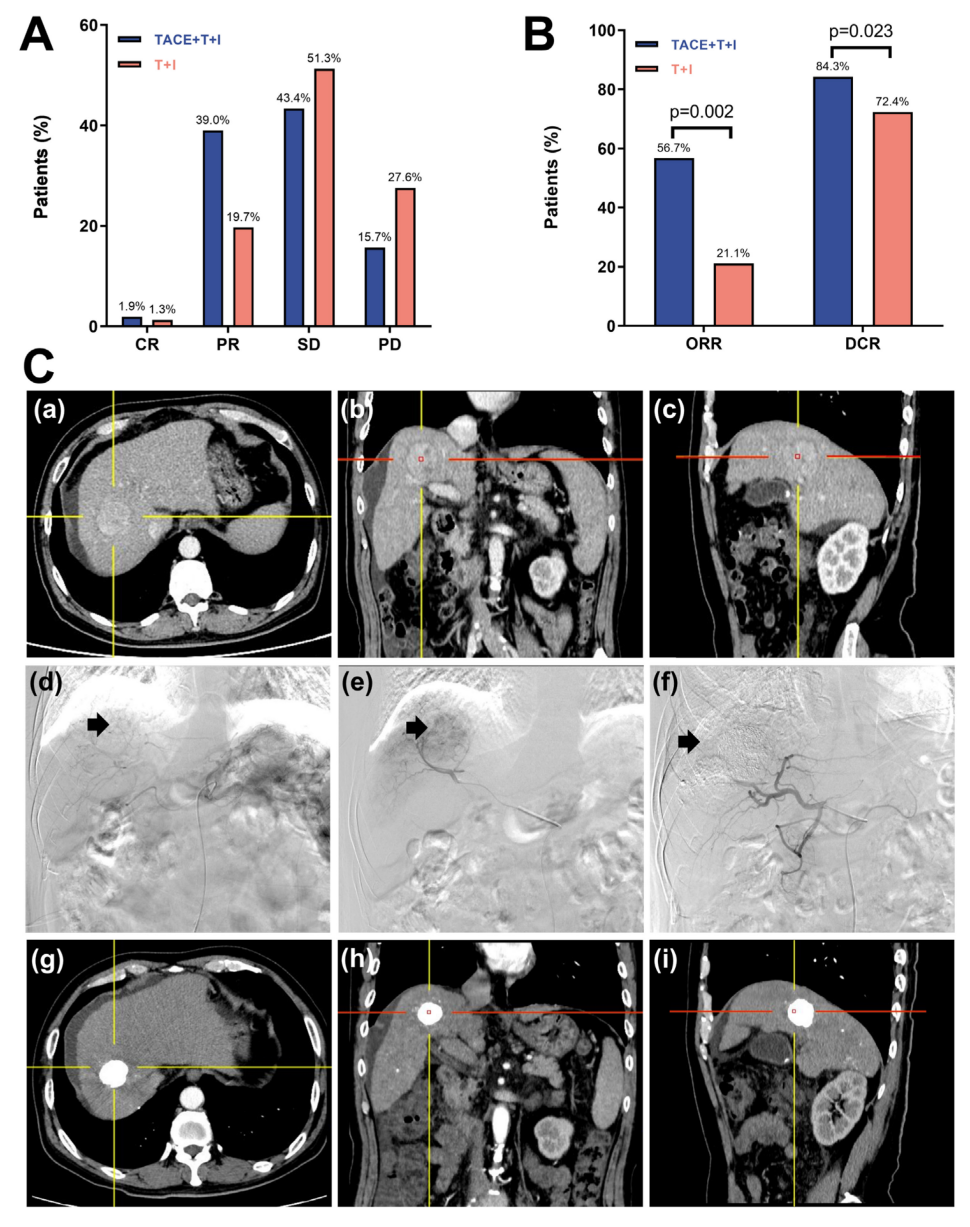
Tumor Response Assessment of patients receiving ICI and TKI treatment with/without TACE. (A, B) histograms of tumor response in Unresectable HCC Assessed by mRECIST Criteria. (C) One case of the TACE+T+I group. The patient was a 58 years old male. (a-c) The axial, coronal, and sagittal images of the contrast-enhanced CT scan showed a tumor in the right lobe of the liver. (d) Hepatic artery angiography showed large tumor staining (arrow head) of the right lobe of the liver. (e) Superselective arterial angiography and embolization of the tumor-feeding artery branches. (f) Angiography after embolization, tumor blood supply was significantly reduced. (g-i) One-year follow-up contrast-enhanced CT scan showed a significant accumulation of iodized oil at the tumor site in the right lobe of the liver, with no obvious enhancement of the tumor. TACE+T+I, transarterial chemoembolization combined with tyrosine kinase inhibitor and immune checkpoint inhibitor group; T+I, tyrosine kinase inhibitor combined with immune checkpoint inhibitor group; TACE, transarterial chemoembolization; ICI, immune checkpoint inhibitor; TKI, tyrosine kinase inhibitor; CR, complete response; PR, partial response; SD, stable disease; PD, progressive disease; ORR, overall response rate; DCR, disease control rate.

The tumor response based on the mRECIST is presented in Table 2 and Fig. 3A, 3B. As shown in Fig. 3A, the percentages of CR and PR were higher in the TACE+T+I group than in the T+I group. Specifically, the CR rates were 1.9% in the TACE+T+I group and 1.3% in the T+I group, while the PR rates were 39.0% and 19.7%, respectively (Fig. 3C). Additionally, as depicted in Fig. 3B, both the ORR and DCR were elevated in the TACE+T+I group. The ORR was 56.7% in the TACE+T+I group and 21.1% in the T+I group (*P* = 0.002), and the DCR was 84.3% and 72.4%, respectively (*P* = 0.023).

### Prognostic factors analysis

Univariate and multivariate cox regression analysis were adopted to assess the risk factors for PFS (Table 3) and OS (Table 4). As shown in Table 3, the univariate analysis of PFS indicated that combination therapy, ECOG PS (1 vs. 0), BCLC stage (B vs. A, C vs. A), AST, TBIL, ALP, PT had P values < 0.10 and were further analyzed through multivariate analysis. The multivariate analysis revealed that combination therapy (TACE+T+I vs. T+I) (HR=0.61; 95% CI: 0.45-0.82; *P* = 0.001) and PT (HR=1.11; 95% CI: 1.01-1.24; *P* = 0.039) were independent predictors of PFS. Similarly, as shown in Table 4, the univariate analysis of OS indicated that combination therapy, age, tumor size, tumor distribution, Child-Pugh classification, cirrhosis, AST, TBIL, ALB, PT, INR, NLR had P values < 0.10. And the multivariate analysis revealed that combination therapy (TACE+T+I vs. T+I) (HR=0.53; 95% CI: 0.39-0.73; *P* < 0.001), age group (≥65 vs. <65) (HR=1.58; 95% CI: 1.04-2.39; *P* = 0.031), tumor size (≥10cm vs. <10cm) (HR=1.36; 95% CI: 1.01-1.84; *P* = 0.043), and PT (HR=1.27; 95% CI: 1.05-1.54; *P* = 0.014) were independent predictors of OS.

**Figure 4:**
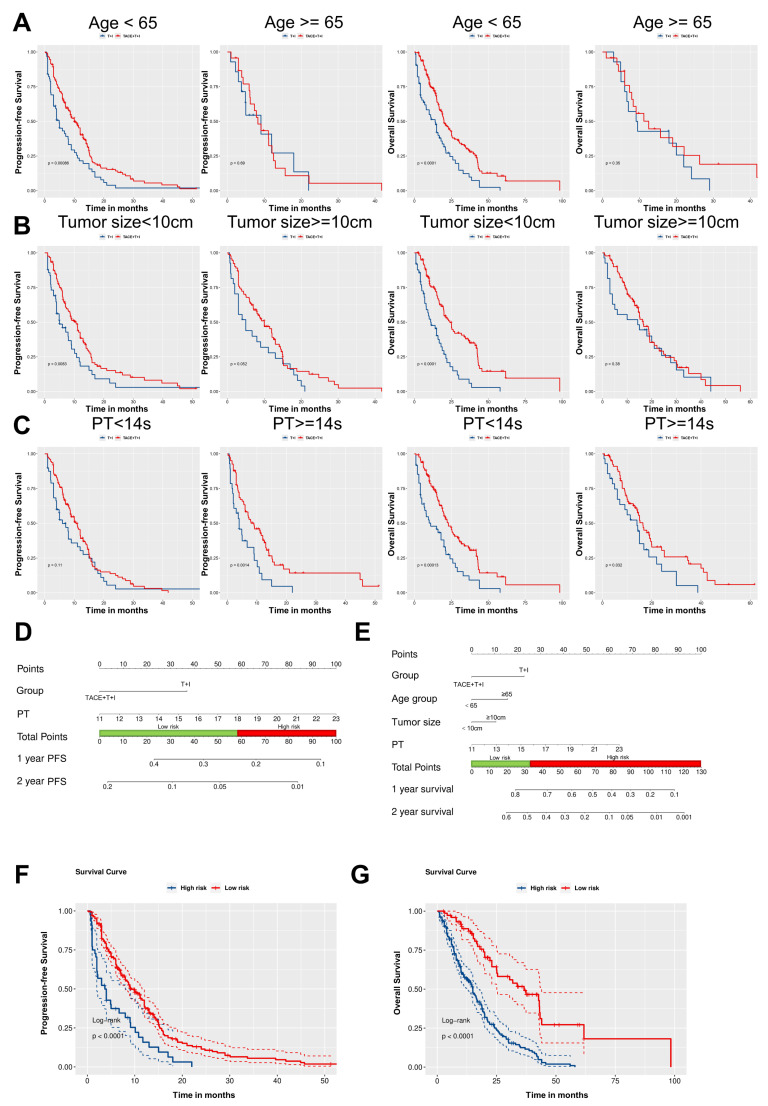
Development of Independent Prognostic Predictors and Subgroup Analysis. (A-C) Kaplan-Meier curves for progression-free survival (PFS) and overall survival (OS) in the TACE+T+I and T+I groups, stratified by age (<65 years or ≥65 years) (A), tumor size (<10 cm or ≥10 cm) (B), and prothrombin time (PT <14s or ≥14s) (C). (D, E) Nomogram of the predictive model for PFS and OS based on Cox multivariate analysis. (F, G) Kaplan-Meier survival curves for risk stratification in the cohort PFS and OS, respectively. TACE+T+I, transarterial chemoembolization combined with tyrosine kinase inhibitor and immune checkpoint inhibitor group; T+I, tyrosine kinase inhibitor combined with immune checkpoint inhibitor group; TACE, transarterial chemoembolization; ICI, immune checkpoint inhibitor; TKI, tyrosine kinase inhibitor; PT, prothrombin time.

As illustrated in Fig. 4A-C, subgroup analyses based on patients' age, tumor size, and PT revealed that the TACE+T+I group achieved significantly better PFS and OS compared to the T+I group across several subgroups. Specifically, in patients aged <65 years, the TACE+T+I group demonstrated superior PFS (*P* = 0.0009) and OS (*P* < 0.0001). Similarly, in patients with tumor size <10 cm, the TACE+T+I group had better PFS (*P* = 0.0083) and OS (*P* < 0.0001). Additionally, in patients with PT >14s, the TACE+T+I group exhibited improved PFS (*P* = 0.0014) and OS (*P* = 0.032). Moreover, as the BCLC staging system is a crucial indicator for patient risk stratification in clinical practice, we conducted a subgroup analysis by dividing patients into two subgroups according to BCLC stages A/B and C. As demonstrated in Fig. S1, the TACE+T+I group exhibited significantly superior OS compared to the T+I group across both subgroups, while demonstrated a significantly prolonged PFS in patients with BCLC stage C.

Based on the results of the multivariate analysis, we constructed nomograms for PFS and OS respectively, as shown in Fig. 4D and 4E. Using the Youden index of the total score as the cutoff value, we divided patients into low-risk and high-risk groups. For PFS, a total score below 58.6 was considered low-risk, and above 58.6 was considered high-risk. The median PFS for the low-risk cohort was 8.0 months (IQR: 4.0-13.0 months), compared to 3.7 months (IQR: 1.5-8.1 months) for those in the high-risk group, with a statistically significant difference observed (Log-rank *P* < 0.0001; Fig. 4F). The estimated median OS for the low-risk group was 21.2 months (IQR: 13.6-37.2 months), while for the high-risk group, it was 11.2 months (IQR: 6.0-19.0 months) (Log-rank *P* < 0.0001; Fig. 4G).

### Adverse events

As shown in Table 5, the adverse events related to TACE, ICI, and TKI were recorded. There were no treatment-related deaths. The most common adverse events in both the TACE+T+I and T+I groups were appetite loss (37.6% and 40.4%, respectively), hand-foot skin reaction (29.5% and 31.6%, respectively), hypertension (19.5% and 25.0%, respectively), diarrhea (17.6% and 26.3%, respectively), and pain (15.7% and 21.1%, respectively). Most AEs were grade 1-2, with rare occurrences of grade 3 or higher AEs. These AEs could be well managed with symptomatic treatment.

## Discussion

In this study, we retrospectively included patients with advanced HCC who received either TACE combined with TKI and ICI, or TKI and ICI alone. We found that: (1) compared to TKI and ICI therapy, the combination of TACE with TKI and ICI showed significant survival benefits in HCC patients, with longer PFS, OS, and higher ORR, DCR; (2) In addition to treatment strategies, PT was also an independent predictor of both PFS and OS; (3) the nomogram, constructed based on independent predictors, can accurately stratifies patients into high-risk and low-risk groups, thereby facilitating medical decision-making and improving prognosis; (4) The combination of TACE and systemic therapy was generally well-tolerated and associated with controllable AEs.

This study distinguishes itself from others by incorporating a broader multicenter patient dataset and a more extensive array of clinical variables and laboratory characteristics, which undoubtedly enhances the reliability and robustness of the results. Furthermore, by constructing a nomogram, we quantified the independent predictors selected in the multivariate Cox analysis, allowing us to predict the 1-year and 2-year survival rates of patients. Based on the nomogram's predictive results, we can accurately divide patients into high-risk and low-risk groups, thus providing more precise guidance for clinical intervention.

Previous research has shown that TACE treatment induces substantial alterations in the tumor microenvironment, including the development of hypoxia and an increase in vascular endothelial growth factor (VEGF) levels 22,27-29. These changes promote angiogenesis and tumor progression. Therefore, combining TACE with TKI therapy targeting VEGF may offer enhanced therapeutic benefits. At the same time, TACE-induced tumor cell death releases tumor antigens, enhancing T-cell infiltration into the tumor. This process transforms the initially immunotherapy-resistant "cold" tumor into a "hot" tumor that is more responsive to immunotherapy interventions 20,21,30,31. However, some studies have pointed out that TACE treatment can increase the expression of PD-L1 on tumor cell surfaces, potentially impeding T-cell function and fostering an immunosuppressive tumor microenvironment 32,33. These findings provide theoretical support for the combination of TACE and ICI. Notably, research conducted by Roger Esteban-Fabró et al. revealed that cabozantinib therapy significantly reduces the population of CD8^+^PD-1^+^ T cells within the tumor microenvironment while increasing the infiltration of neutrophils, thus enhancing therapeutic efficacy for patients with HCC 34. This implies that TKI might possess immunomodulatory properties, and when used in conjunction with ICI, could potentially create synergistic effects. Thus, it is conceivable that the approach of administering TKI and ICI following TACE is a rational one, potentially maximizing treatment outcomes.

The results of the multivariate COX analysis indicate that PT is an independent predictor of both PFS and OS, consistent with some previous studies 35-38. As a vital liver function marker, prolonged PT generally indicates a diminished capacity of the liver to produce essential coagulation factors. The liver is responsible for producing most coagulation factors, and liver dysfunction leads to a decrease in the synthesis of these proteins, impacting the patient's coagulation capabilities. Poor liver function in patients heightens the risk of bleeding and thrombosis, which is detrimental to the prognosis of patients with HCC. Additionally, the multivariate COX analysis found that age ≥65 years and tumor size ≥10cm are risk factors for OS 39,40. Older patients generally have poorer overall conditions and limited anti-tumor capabilities, leading to worse survival rates. Larger tumors, especially those ≥10cm (classified as large liver cancer), impose a greater tumor burden to patients, impair liver function, cause metabolic abnormalities, and present earlier with cancer-consuming symptoms such as weight loss and muscle atrophy. Additionally, they impose a greater psychological burden, all of which are detrimental to the overall survival of patients. The subgroup analysis further revealed that for patients with PT ≥ 14 seconds, age < 65 years, and tumor size < 10 cm, incorporating TACE into the combination therapy of targeted therapy and immunotherapy significantly extends PFS and OS, providing greater benefits. This suggests that we should consider TACE treatment at the early stages of disease, as patients at this time have better baseline health status and higher treatment tolerance.

Most AEs in this study were grade 1 or 2, and patients tolerated the treatment well without needing to reduce dosage or suspend treatment. These symptoms gradually resolved within 1 to 2 weeks. The TACE-related post-embolization syndrome is usually transient and self-limiting. The occasional grade 3 AEs could be reduced to grade 1-2 after dose reduction or temporary interruption.

Although this study provides valuable insights, its limitations cannot be ignored. As a retrospective study, it may be subject to selection biases that could affect the generalizability of the results. Moreover, due to the unique epidemiological characteristics of Chinese liver cancer patients, most of participants in this study were male, with HBV infection and cirrhosis, which may partially limit the broader applicability of the results. To address these key issues, future research must include well-designed prospective, multicenter clinical studies to verify the findings of this study and further explore the optimal indications and treatment regimens for combination therapy.

In conclusion, this study confirms that for patients with advanced HCC, the combination of TACE with TKI and ICI significantly improves treatment effects and extends patient survival compared to the treatment of TKI and ICI alone. PT is clinical prognostic factors for both PFS and OS. These results indicate that the integration of local and systemic therapies could emerge as a pivotal treatment approach for patients with advanced HCC.

## Supplementary Material

Supplementary figure.

## Figures and Tables

**Table 1:** Patient characteristics at baseline.

Characteristics	T+I group (*n* = 76)	TACE+T+I group (*n* = 210)	*P*-value
Age group (years) (%)			0.143
<65	62(81.6)	187(89.0)	
≥65	14(18.4)	23(11.0)	
Sex (%)			0.540
Male	63 (82.9)	182 (86.7)	
Female	13(17.1)	28(13.3)	
ECOG PS (%)			0.646
0	31(40.8)	78(37.1)	
1	43(56.6)	129(61.4)	
2	2(2.6)	3(1.4)	
Child-Pugh class (%)			0.519
A	54(71.1)	159(75.7)	
B	22(28.9)	51(24.3)	
BCLC stage (%)			0.176
A	4(5.3)	4(1.9)	
B	14(18.4)	29(13.8)	
C	58(76.3)	177(84.3)	
Liver cirrhosis (%)			0.847
No	66(86.8)	186(88.6)	
Yes	10(13.2)	24(11.4)	
Etiology (%)			0.763
Hepatitis B	59(77.6)	171(81.4)	
Hepatitis C	4(5.3)	10(4.8)	
Non-B, non-C	13(17.1)	29(13.8)	
Tumor distribution (%)			0.296
Single	17(22.4)	62(29.5)	
Multiple	59(77.6)	148(70.5)	
Tumor size (%)			0.294
<10 cm	49(64.5)	119(56.7)	
≥10 cm	27(35.5)	91(43.3)	
Laboratory parameters			
RBC (10^9^/L, mean ± SD)	4.21 ± 0.70	4.12 ± 0.71	0.352
Hb (g/L, mean ± SD)	128.79 ± 17.77	124.77 ± 21.87	0.151
Platelet (10^9^/L, mean ± SD)	179.49 ± 77.81	177.92 ± 97.18	0.900
WBC (10^12^/L, mean ± SD)	6.05 ± 2.24	6.08 ± 2.36	0.934
Neutrophils (10^9^/L, mean ± SD)	3.98 ± 2.08	4.65 ± 6.09	0.352
Lymphocyte (10^9^/L, mean ± SD)	1.46 ± 2.00	1.40 ± 1.91	0.804
NLR (mean ± SD)	3.94 ± 2.95	4.53 ± 4.79	0.316
ALT (U/L, mean ± SD)	54.69 ± 53.31	63.79 ± 71.42	0.312
AST (U/L, mean ± SD)	80.84 ±86.63	78.93 ± 99.56	0.882
TBIL (mmol/L, mean ± SD)	21.97 ± 21.75	21.62 ± 20.32	0.900
ALP (U/L, mean ± SD)	173.68 ± 105.53	168.61 ± 111.52	0.731
TBA (mmol/L, mean ± SD)	20.93 ± 25.60	16.16 ± 28.52	0.201
TP (g/L, mean ± SD)	65.78 ±7.16	64.22 ±7.08	0.103
Cr (μmol/L, mean ± SD)	66.82 ±14.47	69.09 ±46.51	0.677
UA (μmol/L, mean ± SD)	296.20 ±76.94	293.38 ±105.45	0.831
ALB (g/L, mean ± SD)	36.80 ± 5.79	36.39 ± 5.28	0.566
PT (s, mean ± SD)	13.89 ±1.19	13.87 ±1.31	0.925
INR (mean ± SD)	1.11 ±0.10	1.10 ±0.13	0.673
AFP (ng/ml) (%)			0.859
<400	45(59.2)	120(57.1)	
≥400	31(40.8)	90(42.9)	

Data are mean±SD or N (%). TACE, transarterial chemoembolization; T+I, tyrosine kinase inhibitor (TKI) plus immune checkpoint inhibitor (ICI); TACE+T+I, TACE plus TKI plus ICI; ECOG PS, Eastern Cooperative Oncology Group Performance Status; BCLC, Barcelona Clinic Liver Cancer; RBC, red blood cell; Hb, hemoglobin; WBC, white blood cell; NLR, neutrophil-to-lymphocyte ratio; ALT, alanine aminotransferase; AST, aspartate aminotransferase; TBIL, total bilirubin; ALP, alkaline phosphatase; TBA, total bile acid; TP, total protein; Cr, creatinine; UA, uric acid; ALB, albumin; PT, prothrombin time; INR, international normalized ratio; AFP, alpha-fetoprotein.

**Table 2:** Treatment efficacy evaluated by mRECIST criteria.

	T+I group (n=76)	TACE+T+I group (n=210)	P-value
Tumor response			0.001
CR	1(1.3)	4(1.9)	
PR	15(19.7)	82(39.0)	
SD	39(51.3)	91(43.3)	
PD	21(27.6)	33(15.7)	
ORR	16(21.1)	86(56.7)	0.002
DCR	55(72.4)	177(84.3)	0.023

Values are presented as N (%). mRECIST, modified response evaluation criteria in solid tumours; TACE, transarterial chemoembolization; CR, complete response; PR, partial response; SD, stable disease; PD, progressive disease; ORR, overall response rate; DCR, disease control rate.

**Table 3:** Results of the univariable and multivariable Cox regression result of PFS.

	Univariate			Multivariate		
Characteristics	HR	95%CI	P-value	HR	95%CI	P-value
Group (TACE+T+I group vs. T+I group)	0.63	0.47-0.85	0.002	0.61	0.45-0.82	0.001
Age	1.01	0.99-1.02	0.367			
Age group (≥65 vs. <65)	1.11	0.75-1.63	0.605			
Gender (Female vs. Male)	0.85	0.58-1.25	0.402			
ECOG PS						
1 vs. 0	1.38	1.05-1.83	0.023	-		
2 vs. 0	2.16	0.79-5.95	0.135			
BCLC stage						
B vs. A	2.93	1.01-8.50	0.048	-		
C vs. A	3.28	1.18-9.13	0.023	-		
Tumor size (≥10cm vs. <10cm)	1.11	0.85-1.44	0.456			
Tumor distribution (multiple vs. single)	1.01	0.75-1.36	0.952			
Child-Pugh class (B vs. A)	1.27	0.93-1.72	0.133			
Liver cirrhosis (yes vs. no)	0.98	0.63-1.51	0.927			
HBV (yes vs. no)	0.89	0.64-1.24	0.488			
AFP (≥400 vs. <400)	1.13	0.87-1.47	0.347			
Laboratory parameters						
RBC	1.04	0.88-1.23	0.671			
WBC	0.99	0.94-1.06	0.848			
Platelet	1.00	1.00-1.00	0.293			
Hb	1.00	1.00-1.01	0.463			
Neutrophils	0.99	0.95-1.03	0.484			
Lymphocyte	1.02	0.94-1.11	0.651			
ALT	1.00	1.00-1.00	0.117			
AST	1.00	1.00-1.00	0.044	-		
TBIL	1.01	1.00-1.01	0.029	-		
ALP	1.00	1.00-1.00	0.010	-		
TBA	1.00	1.00-1.00	0.854			
TP	1.00	0.98-1.02	0.858			
Cr	1.00	0.99-1.00	0.376			
UA	1.00	1.00-1.00	0.130			
ALB	0.98	0.96-1.00	0.118			
PT	1.11	1.00-1.22	0.053	1.11	1.01-1.24	0.039
INR	2.02	0.63-6.42	0.235			
NLR	1.01	0.99-1.04	0.365			

PFS, Progression-Free Survival; HR, Hazard Ratio; CI, Confidence Interval; TACE, transarterial chemoembolization; T+I, tyrosine kinase inhibitor (TKI) plus immune checkpoint inhibitor (ICI); TACE+T+I, TACE plus TKI plus ICI; ECOG PS, Eastern Cooperative Oncology Group Performance Status; BCLC, Barcelona Clinic Liver Cancer; HBV, Hepatitis B Virus; RBC, red blood cell; Hb, hemoglobin; WBC, white blood cell; NLR, neutrophil-to-lymphocyte ratio; ALT, alanine aminotransferase; AST, aspartate aminotransferase; TBIL, total bilirubin; ALP, alkaline phosphatase; TBA, total bile acid; TP, total protein; Cr, creatinine; UA, uric acid; ALB, albumin; PT, prothrombin time; INR, international normalized ratio; AFP, alpha-fetoprotein.

**Table 4:** Results of the univariable and multivariable Cox regression result of OS.

	Univariate			Multivariate		
Characteristics	HR	95%CI	P-value	HR	95%CI	P-value
Group (TACE+T+I group vs. T+I group)	0.52	0.39-0.71	<0.001	0.53	0.39-0.73	<0.001
Age	1.00	0.99-1.02	0.478			
Age group (≥65 vs. <65)	1.73	1.17-2.56	0.006	1.58	1.04-2.39	0.031
Gender (Female vs. Male)	0.89	0.59-1.34	0.565			
ECOG PS						
1 vs. 0	1.11	0.83-1.48	0.481			
2 vs. 0	1.05	0.38-2.86	0.927			
BCLC stage						
B vs. A	1.47	0.51-4.20	0.475			
C vs. A	2.04	0.75-5.53	0.164			
Tumor size (≥10cm vs. <10cm)	1.44	1.08-1.92	0.014	1.36	1.01-1.84	0.043
Tumor distribution (multiple vs. single)	1.32	0.95-1.83	0.100			
Child-Pugh class (B vs. A)	1.35	0.98-1.88	0.070	-		
Liver cirrhosis (yes vs. no)	0.57	0.34-0.97	0.038	-		
HBV (yes vs. no)	1.09	0.76-1.56	0.641			
AFP (≥400 vs. <400)	1.09	0.82-1.45	0.536			
Laboratory parameters						
RBC	0.96	0.78-1.18	0.703			
WBC	1.02	0.96-1.08	0.500			
Platelet	1.00	1.00-1.00	0.647			
Hb	1.00	0.99-1.00	0.181			
Neutrophils	1.00	0.97-1.04	0.821			
Lymphocyte	1.00	0.90-1.10	0.932			
ALT	1.00	1.00-1.00	0.339			
AST	1.00	1.00-1.00	0.039	-		
TBIL	1.01	1.00-1.01	0.042	-		
ALP	1.00	1.00-1.00	0.053	-		
TBA	1.00	0.99-1.00	0.572			
TP	1.00	0.98-1.02	0.671			
Cr	1.00	1.00-1.00	0.621			
UA	1.00	1.00-1.00	0.661			
ALB	0.97	0.94-0.99	0.004	-		
PT	1.16	1.04-1.28	0.006	1.27	1.05-1.54	0.014
INR	3.03	1.00-9.17	0.050	-		
NLR	1.03	1.00-1.05	0.030	-		

OS, Overall Survival; HR, Hazard Ratio; CI, Confidence Interval; TACE, transarterial chemoembolization; T+I, tyrosine kinase inhibitor (TKI) plus immune checkpoint inhibitor (ICI); TACE+T+I, TACE plus TKI plus ICI; ECOG PS, Eastern Cooperative Oncology Group Performance Status; BCLC, Barcelona Clinic Liver Cancer; HBV, Hepatitis B Virus; RBC, red blood cell; Hb, hemoglobin; WBC, white blood cell; NLR, neutrophil-to-lymphocyte ratio; ALT, alanine aminotransferase; AST, aspartate aminotransferase; TBIL, total bilirubin; ALP, alkaline phosphatase; TBA, total bile acid; TP, total protein; Cr, creatinine; UA, uric acid; ALB, albumin; PT, prothrombin time; INR, international normalized ratio; AFP, alpha-fetoprotein.

**Table 5:** Treatment-related adverse events.

Adverse Events	T+I group (n=76)		TACE+T+I group (n=210)	
	All grade	Grade≥3	All grade	Grade≥3
Hypertension	19(25.0)	2(2.6)	41(19.5)	5(2.4)
Nausea and vomiting	6(7.9)	0(0.0)	11(5.2)	1(0.5)
Pain	16(21.1)	3(3.9)	33(15.7)	1(0.5)
Hand-foot skin reactions	24(31.6)	1(1.3)	62(29.5)	13(6.2)
Diarrhea	20(26.3)	0(0.0)	37(17.6)	2(1.0)
RCCEP	7(9.2)	0(0.0)	13(6.2)	1(0.5)
Fatigue	19(25.0)	1(1.3)	39(18.6)	3(1.4)
Decreased appetite	31(40.8)	5(6.6)	79(37.6)	4(1.9)
Pneumonitis	3(3.9)	0(0.0)	9(4.3)	0(0.0)
Hypothyroidism	0(0.0)	0(0.0)	3(1.4)	0(0.0)
Rash	2(2.6)	0(0.0)	5(2.4)	0(0.0)
Hemorrhage	2(2.6)	0(0.0)	8(3.8)	0(0.0)

Values are presented as N (%). TACE, transarterial chemoembolization; T+I, tyrosine kinase inhibitor (TKI) plus immune checkpoint inhibitor (ICI); TACE+T+I, TACE plus TKI plus ICI; RCCEP: Reactive Cutaneous Capillary Endothelial Proliferation.

## Abbreviations

TACE: transarterial chemoembolization
TKI: tyrosine kinase inhibitor
ICI: immune checkpoint inhibitor
HCC: hepatocellular carcinoma
PFS: progression-free survival
OS: overall survival
ORR: objective response rate
DCR: disease control rate
PT: prothrombin time
BCLC: Barcelona Clinic Liver Cancer
PD-1: programmed death protein 1
ECOG: Eastern Cooperative Oncology Group
HBV: hepatitis B virus
RBC: red blood cell
WBC: white blood cell
Hb: hemoglobin
PLT: platelet
TBIL: total bilirubin
ALB: albumin
ALT: alanine aminotransferase
AST: aspartate aminotransferase
ALP: alkaline phosphatase
Cr: creatinine
UA: uric acid
TBA: total bile acid
INR: international normalized ratio
mRECIST: modified Response Evaluation Criteria in Solid Tumors
CR: complete response
PR: partial response
SD: stable disease
PD: progressive disease
AE: adverse event
IQR: interquartile range
HR: hazard ratio
VEGF: vascular endothelial growth factor
