# Supplementary material for: TACE Empowers Immune Checkpoint Inhibitors and Tyrosine Kinase Inhibitors in Unresectable HCC: A Multicenter Retrospective Study
Source: J Cancer. 2025 Jun 12;16(8):2750–61. doi: 10.7150/jca.112706 (PMC12170989; doi:10.7150/jca.112706)
Supplement: Supplementary file 1 — Supplementary figure. [file jcav16p2750s1.pdf]

Supplementary Figures

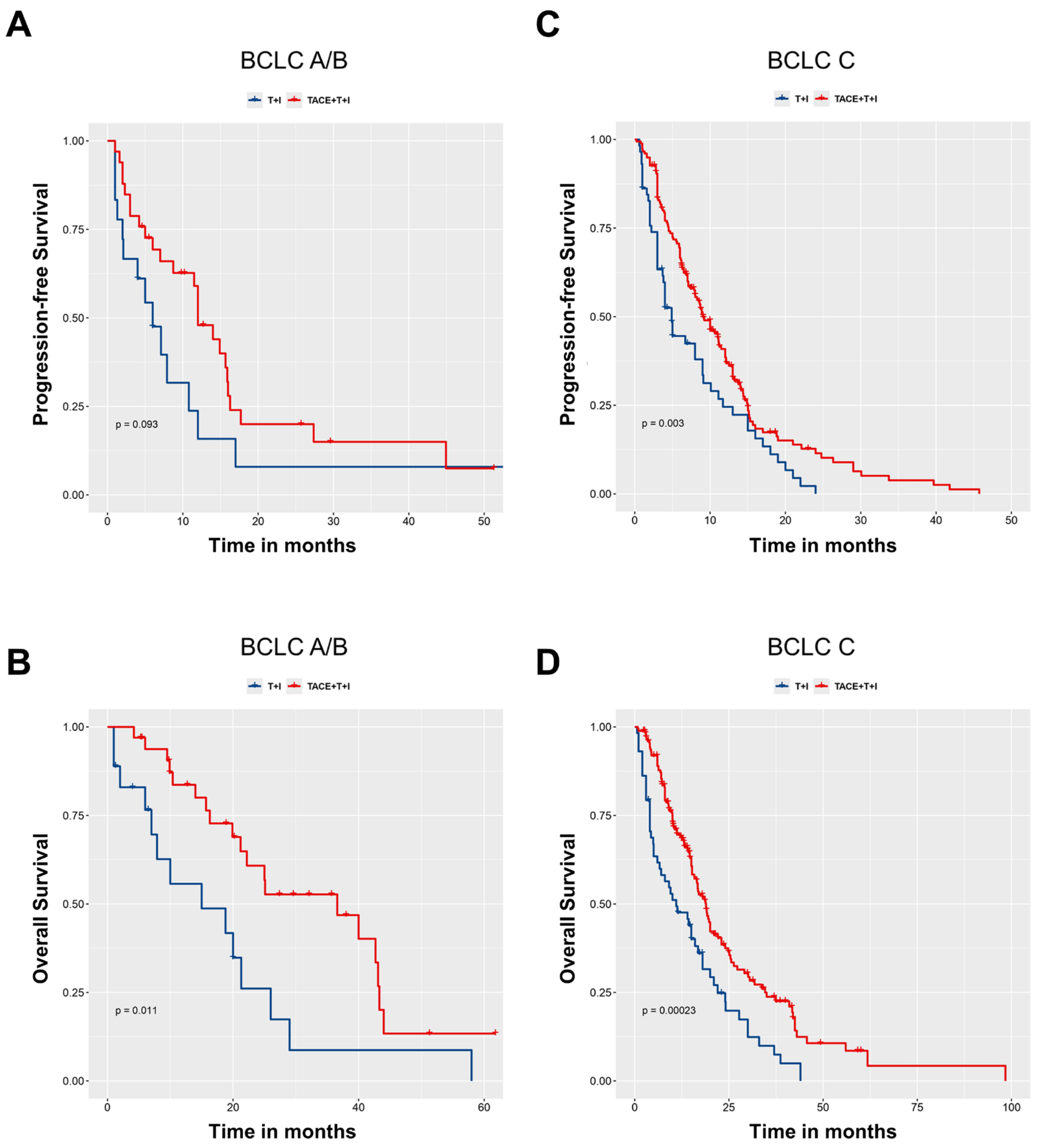

Fig. S1 Subgroup analysis based on BCLC staging system. (A-D) Kaplan-Meier curves for progression-free survival (PFS) and overall survival (OS) in the TACE+T+I and T+I groups, stratified by BCLC staging system (A/B stage or C stage). TACE+T+I, transarterial chemoembolization combined with tyrosine kinase inhibitor and immune checkpoint inhibitor group; T+I, tyrosine kinase inhibitor combined with immune checkpoint inhibitor group; TACE, transarterial chemoembolization; ICI, immune checkpoint inhibitor; TKI, tyrosine kinase inhibitor.
